# P52 Predictors and sources of antibiotic consumption in The Gambia

**DOI:** 10.1093/jacamr/dlaf118.059

**Published:** 2025-07-14

**Authors:** Abigail Lawrence, Stephen Owens, Phoebe Weddle, Matthew Breckons, Karen Forrest, Behzad Nadjm

**Affiliations:** Newcastle University, Newcastle, UK; Newcastle University, Newcastle, UK; Newcastle University, Newcastle, UK; Newcastle University, Newcastle, UK; MRC Unit The Gambia, LSHTM, Serrekunda, The Gambia; LSHTM, London, UK

## Abstract

**Objectives:**

To identify the sources of antibiotics consumed by households and to identify predictors of antibiotic consumption.

**Methods:**

A survey was conducted with 140 households, identified by a spatial sampling frame using randomly generated GPS coordinates and satellite imaging. The questionnaire was adapted from a validated WHO household survey tool. A ‘drugs bag’, containing samples of all antibiotics available within the region, was created to aid recall during the questionnaire. This included different packing, names, and formulations (except injectables). Multivariate binary logistic regression was used to analyse potentials predictor values of antibiotic consumption.

**Results:**

In total, 71.4% of households surveyed reported an acute illness episode within the last 6 months, of which 84% consumed medication and 45% consumed antibiotics. The most common source of antibiotics was the pharmacy (44%).

**Conclusions:**

Initial antimicrobial resistance strategies implemented in The Gambia could be aimed at pharmacies to maximize impact. None of the expected predictor values showed significance for antibiotic consumption. Further studies considering new variables or with a larger sample size may elicit statistically significant predictors.

